# Severe Hypertriglyceridemia: A 10-Year Review in a Portuguese Hospital

**DOI:** 10.7759/cureus.41239

**Published:** 2023-06-30

**Authors:** Francisco S Laranjeira, Nuno M Neves, Anabela Raimundo, Alexandra Bayão Horta

**Affiliations:** 1 Internal Medicine Department, Hospital da Luz Lisboa, Lisboa, PRT; 2 Internal Medicine Department, Hospital Da Luz Lisboa, Lisboa, PRT

**Keywords:** fibrates, therapeutic plasmapheresis, cardiovascular risk (cvr), hypertriglyceridemia-induced acute pancreatitis, severe hypertriglyceridemia

## Abstract

Introduction: Severe hypertriglyceridemia (SHTG) is a rare condition associated with serious complications, such as acute pancreatitis (AP), and the best treatment is still a matter of discussion. The aim of this study is to outline the demographics, management, and outcomes (recurrence and mortality) of complications in patients with SHTG.

Material and methods: A retrospective, observational, and analytical study was carried out by obtaining clinical data from the electronic health records of patients with SHTG admitted to the Internal and Intensive Medicine units from the 1^st^ of January 2009 to the 31^st^ of December 2020 in a university hospital.

Results: The cohort included 17 patients. The most common complication was AP (13/17 = 76.5%). Admission to the intensive care unit (ICU) was observed in 84.2%. Among patients with AP, the most commonly administered therapies were insulin (82.4%) and fibrates (76.5%). Plasmapheresis was used in 58.8%, and the criteria for using this technique were mainly based on clinical and laboratory abnormalities. There were no deaths. The readmission rate at 30 days was 36.3%.

Conclusion: This study shows the morbidity profile associated with SHTG, with a high level of ICU admissions and also a high level of the use of plasmapheresis. In our population, this approach had good results, and this should be highlighted as there are no clear international guidelines for this intervention. Distinguishing between patients with familial chylomicronemia syndrome or with multifactorial chylomicronemia is important as recent specific therapy for lipoprotein lipase (LPL) genetic deficit is available. In the near future, the performance of a genetic study should be considered in patients with SHTG as an attempt to avoid the high recurrence rate of complications of this disease.

## Introduction

Severe hypertriglyceridemia (SHTG) is defined as a serum triglyceride (TG) level > 1000 mg/dL [1]. It is a rare condition that may be associated with many complications, some of which are severe, such as acute pancreatitis [2].

The etiology of hypertriglyceridemia (HTG) can be divided into primary and secondary [1]. Primary HTG causes more severe HTG, although it is the interplay of both primary and secondary factors that leads to SHTG [3]. Severe HTG is most commonly seen with three conditions: familial chylomicronemia syndrome (FCS), primary HTG, and mixed HTG. FCS and mixed HTG lead to more SHTG, and the complications are more premature clinically, whereas primary HTG is most frequently diagnosed in adulthood and precipitated by secondary factors. The most common genetic defects leading to severe HTG include LPL deficiency or gene mutation, apolipoprotein C II deficiency, and mutations in other genes involved in lipoprotein generation and metabolism. It is recognized that the etiology of HTG is complex and very challenging to treat, especially because hypertriglyceridemia is more often polygenic in nature and only rarely constitutes a monogenic disorder [4]. When HTG is severe, it is most likely to have a monogenic defect in contrast to mild to moderate HTG, which is more likely to have a polygenic defect. Secondary factors include alcohol abuse, obesity, hypothyroidism, diabetes mellitus, chronic renal failure, and the use of drugs like estrogen or corticosteroids. A detailed anamnesis to elicit the underlying factors in patients with hypertriglyceridemia-acute pancreatitis (HTG-AP) should be performed, and genetic screening should be considered based on their family history [5].

SHTG can be responsible for significant complications, and AP is definitely the most important one. Being the most significant complication, AP is a serious disease that can cause death or multiple long-term morbidities, such as diabetes mellitus or chronic pancreatitis [1]. After gallstones and alcohol, SHTG is the third most common cause of AP. There is a risk of AP if the triglyceride (TG) level is higher than 500 mg/dL; however, the frequency of pancreatitis is much higher when the level exceeds 1000 mg/dL [2]. Data from European population studies show the incidence of AP between 10% and 19% in patients with severe HTG (>1000 mg/dL) [6].

The role of triglycerides (TG) in the development of cardiovascular disease is still controversial, even more than 60 years after Moreton first postulated a relationship between them. [3]. However, more recent data on SHTG are likely to settle this debate by establishing a consistent relationship between TG level (especially non-fasting TG level) and residual cardiovascular risk. [7]. Besides AP, there are some other complications associated with severe HTG, not so frequent nor so severe, including eruptive xanthomata, lipemia retinalis, neuropathy, and hepatosplenomegaly [8].

Conventional management of SHTG includes fat dietary restriction and pharmacological treatment; however, the slow action of antihyperlipidemic therapy poses a problem when the situation requires a rapid reduction of the levels, and the safety and efficacy of individual treatment options are still lacking [9]. Plasmapheresis (PF) seems to be an efficient and safe therapy when a rapid reduction of triglycerides (TG) is needed as it allows it in just a few hours. PF does not interfere with TG production; therefore, it should be considered as an adjunctive therapy to medical treatment. [10]. Its use has been increasingly reported, especially in high-risk patients admitted to intensive care units. Unfortunately, there are no controlled or randomized studies to prove its benefit in mortality reduction [11]. Most of the data are based on single case reports, and this is the main reason why the American Society for Apheresis (ASFA) recommends the use of PF in the treatment of SHTG complicated with AP as a grade 2C recommendation [12].

This study aimed to outline the demographics, cardiovascular risk factors, treatment, and outcome (recurrence and mortality) in a cohort of patients admitted to a university hospital with SHTG and associated complications, mainly AP.

## Materials and methods

Study design and population

A retrospective, observational, and analytical study was carried out through data collection from the clinical files of patients with SHTG admitted to the Internal and Intensive Medicine units between 1st January 2009 and 31st December 2020 at a university hospital in Lisbon-Portugal. Patients with TGs < 1000 mg/dL, age< 18 years, and incomplete data were excluded. The selected patients were all clinically followed.

Variables

Demographic characteristics of patients were evaluated and included age, sex, race, selected comorbid conditions, risk factors for AP and cardiovascular diseases, concomitant medication use, and laboratory data.

Risk factors (for AP and for cardiovascular diseases) included a history of alcohol abuse, a history of pancreatitis (defined based on the International Classification of Diseases, ninth revision, clinical modification codes), diabetes mellitus, disorders of lipid metabolism, smoking history, and arterial hypertension. Body mass index (BMI) was registered and categorized into three groups: normal, overweight, and obese. Laboratory data collected at admission, after plasmapheresis and at discharge, consisted of total cholesterol (TC) levels, low-density lipoprotein cholesterol (LDL) levels, high-density lipoprotein cholesterol (HDL) levels, triglycerides, and fasting hemoglobin A1C (Hgb A1C) levels. Other clinical information was collected including criteria for systemic inflammatory response syndrome (SIRS), hemodynamic instability, renal dysfunction, metabolic acidosis, and Acute Physiology and Chronic Health Evaluation (APACHE) score.

Regarding the treatment, patients who underwent plasmapheresis were identified, and the number of sessions performed was registered. The use of lipid-lowering therapies was also registered. Readmissions, morbidity (main complications), and mortality were analyzed.

Statistical analysis

Data were analyzed using the SPSS® statistics program (version 28; IBM Corp., Armonk, NY). Given the small size sample, the study is purely descriptive and does not allow for comparisons. The frequencies of the different variables analyzed are expressed in absolute values and percentages.

## Results

The cohort included 17 patients using our selection criteria, with a mean age of 42.4 years and a median of 37.0 years (maximum = 63 and minimum = 27), and 47.1% (n = 8) were women. The average days of hospitalization were 12.41 days, and the mean stay at the ICU was 3.24 days. About 84.2% of the patients required admission to the ICU.

**Figure 1:**
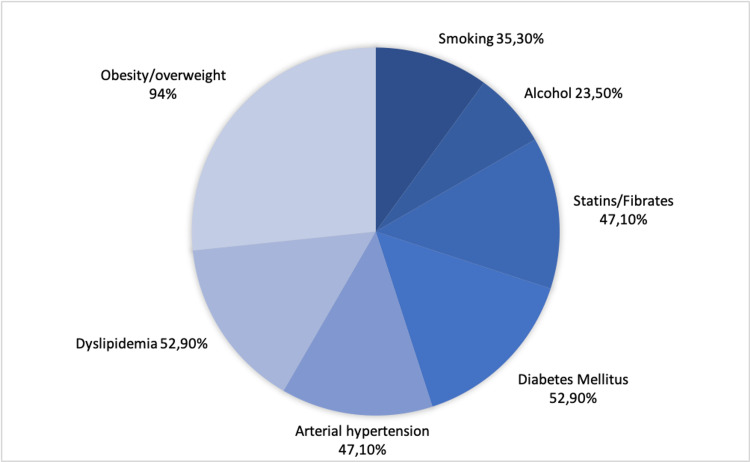
Comorbidities and cardiovascular risk factors

Regarding comorbidities and cardiovascular risk factors, 94% were overweight, of which 18.7% were obese with a BMI > 30 kg/m^2^; 23.5% had excessive alcohol consumption, and 35.3% were active smokers. Moreover, 52.9% had previous dyslipidemia under oral therapy (statin, ezetimibe, or a combination of both), 47.1% had arterial hypertension, and 52.9% had type 2 diabetes mellitus. Figure 1 summarizes baseline patient and clinical characteristics.

Concerning the metabolic profile, the mean triglycerides at admission was 6551.2 mg/dL (minimum = 1063 mg/dL and maximum = 42,340 mg/dL), mean TC of 411.7 mg/dL, and HDL of 30.7 mg/dL. LDL at admission was not calculated as the Friedewald formula does not apply to triglycerides > 400 mg/dL. Regarding glycemic profile, the mean value of HbA1c was 6.28% (maximum = 14.5% and minimum = 5.0%). In the two patients with HbA1c > 10%, the diagnosis of diabetes mellitus was inaugural and presented as an acute decompensation of the disease. Of the seven patients with known diabetes, only three had HbA1c < 8.0% and four had poor metabolic control despite antidiabetic therapy, including insulin. Table 1 summarizes baseline laboratory data including glycemic indices and lipid parameters.

**Table 1:** Glycemic indices and lipid parameters at admission HDL: High-density lipoprotein.

Metabolic Profile	Minimum	Maximum	Mean Value
Triglycerides (mg/dL)	1063	42340	6551
Total cholesterol (mg/dL)	167	1512	412
HDL (mg/dL)	6	62	31
HbA1c (%)	5.0	14.5	6.3

Considering concomitant medications that can change lipid metabolism, seven (41,2%) patients were on antipsychotic medications, two patients were on beta blockers (11.8%), one was on corticosteroids (5.8%), and three on oral estrogens (17.7%).

**Figure 2:**
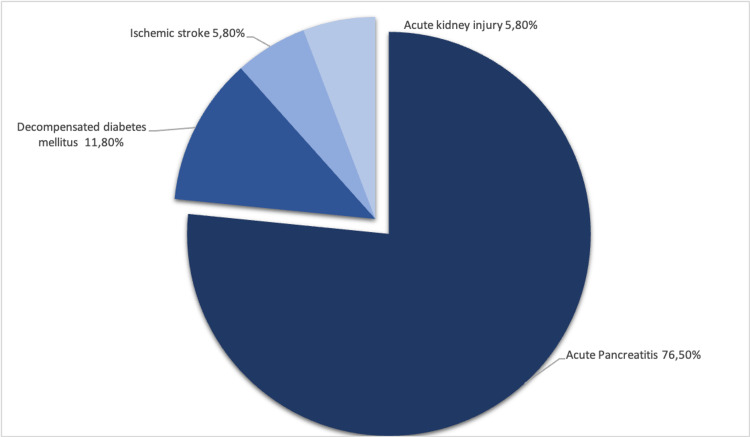
Most common complications

The most common complication was AP, occurring in 13 patients (76.5%), followed by decompensated diabetes mellitus (n = 2), ischemic stroke (n = 1), and acute kidney failure (n = 1). Figure 2 summarizes all complications documented. In the subgroup of patients with AP, there were no patients with, gallstones or alcoholism. Among these patients, the most common therapeutic measures were insulin (82.4%) and fibrates (76.5%), and only 11.8% initiated omega-3 fatty acids. Concerning fibrates, bezafibrate was the most used in about 47.1% of patients (n = 8), followed by fenofibrate (29.4%) and finally gemfibrozil (5.9%). All patients received non-pharmacological treatment, namely, a personalized hypolipidic diet with the orientation of the nutritionist.

**Figure 3:**
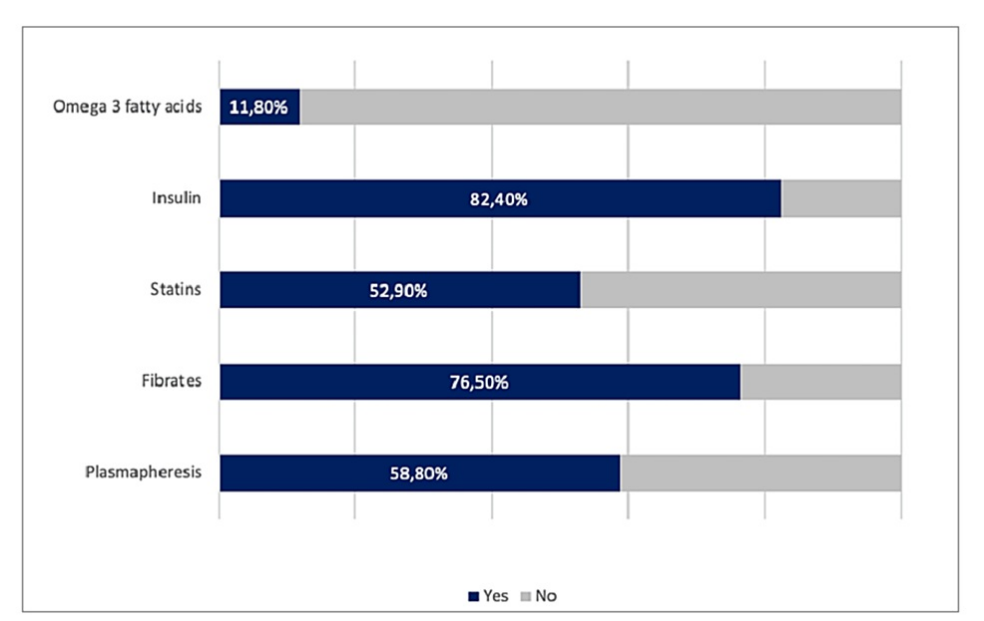
Therapies instituted

Among the patients with SHTG (n = 17), 58.8% (n = 10) required plasmapheresis, in the context of AP. On average, 1.1 sessions were needed for a quick and effective reduction of the triglyceride level, and a single session allowed a reduction of 56.8% in the value of TGs on average. The maximum number of sessions needed was 4 as was the case of a pregnant woman who required an urgent cesarean section in the context of AP associated with SHTG. All therapies instituted are described in Figure 3.

Upon admission to the ICU, the severity assessment score used was the APACHE score with a mean value of 6.3. The criteria for plasmapheresis initiation were either laboratorial, including lactic acidosis (58.8%) and renal dysfunction (23.5%), or clinical, including hemodynamic instability and SIRS that occurred in only 17.6% of patients.

At discharge, mean triglycerides was 564.1 mg/dL. All patients were discharged with a fibrate, and seven of them had associated a statin. There were no deaths. Concerning the follow-up, all of the patients attended a first appointment at the internal medicine outpatient clinic, but only 41.2% maintained follow-up, and the remaining patients either abandoned or maintained follow-up in other specialties such as cardiology or endocrinology (35.3%, n = 6).

The readmission rate was 36.3% (n patients), all were due to AP. All this subset of patients had at least one cardiovascular risk factor, and half of them had at least three factors, with diabetes mellitus being the most common factor. In this group of patients, 83.3% were treated with plasmapheresis in the latest AP episode and hospitalization. Of the 17 patients, three of them suffered a major cardiovascular event, namely, an acute myocardial infarction or stroke after.

## Discussion

In this study, we found 17 patients with SHTG, of whom 70.6% developed AP. This figure is in line with the literature where AP is highly associated with TG > 1000 mg/dL [1]. Our findings are also consistent with those of Toth et al. regarding the clinical characteristics of AP patients, such as hypertension, diabetes, obesity, alcohol abuse, renal disease, and prior history of pancreatitis [13]. In our study, we strengthen the morbidity associated with SHTG, with a high level of admissions to the ICU of 84.2%.

General strategies for the treatment of SHTG include lifestyle modification with cessation of alcohol intake, restriction of dietary fat, and increased exercise. Pharmacologic measures (alone or in combination with drugs) include fibrates (gemfibrozil or fenofibrate), statins, and high-dose omega-3 fatty acids [14]. As a first-line agent, the European Endocrine Society recommends the use of a fibrate for the reduction of TG levels in patients at risk of hypertriglyceridemia-induced AP because fibrates are the most effective drugs in lowering TG levels [15]. In the acute setting, the treatment also includes insulin infusion and fasting. Other effective drugs are omega-3 fatty acids, well tolerated and with a good safety profile, without any serious adverse effects or risk of discontinuation (in respect of the rise in TG values) comparable to the placebo group [16]. In our study, 11.8% of patients initiated omega-3 fatty acids, and all these patients were selected in the most recent years of the cohort. This small number comes from the recent use of omega-3 fatty acids in the outpatient setting (induced by the REDUCE-IT trial) that was maintained in the acute patient [17].

Plasmapheresis is another treatment tool used in the acute setting, especially for patients admitted to the ICU because of SHTG complicated with AP. Published evidence supporting the use of apheresis for HTG-AP is ambiguous, although data suggest its use as early as possible to achieve the best clinical and laboratory results [18]. It is an expensive treatment option, which is not available in all centers, which limits its use, and it has adverse effects that should be considered [19]. Worrisome clinical features in patients with HTG-AP include hypocalcemia, lactic acidosis, signs of worsening systemic inflammation (SIRS), and signs of worsening organ dysfunction or multi-organ failure. More recent case-control studies and a systematic review indicated that while plasmapheresis decreased plasma TG, it did not conclusively reduce the morbidity or mortality of these patients, and it is an expensive therapeutic measure [10]. In our study, we found that plasmapheresis resulted in a significant reduction in TG values, similar to the values achieved in international studies. In our population, the clinical severity was the main criterion for initiation of plasmapheresis, and the approach was effective and should be highlighted as there are no clear international guidelines in this field and more research is needed [20].

The genetic etiology of chylomicronemia may influence the risk of complications such as atherosclerotic diseases and pancreatitis. The balance between risk, benefit, and cost-effectiveness of genetic testing for chylomicronemia should be evaluated [16]. Our data does not allow us to distinguish between patients with multifactorial chylomicronemia or FCS. The recent appearance of specific therapy for LPL genetic deficit (alipogene tiparvovec) may change the pertinence of a genetic study, especially in patients who are more difficult to treat and/or have recurrences [21]. For now, the Consensus Panel report of the European Atherosclerosis Society still does not recommend routine genetic testing for severe HTG [22].

Despite the absence of deaths in our population, it is noteworthy that the rehospitalization rate was considerable, 36% (n = 6). In fact, 50% of the patients who were rehospitalized had previously abandoned outpatient medical follow-up. This raises the hypothesis that this high abandonment of the might be the main reason for high reincidence admissions. Severe HTG-AP has a significantly higher rate of morbimortality when compared to biliary AP and non-HTG etiology, respectively [23]. Furthermore, 50% of the patients from this group of severe HTG-AP had at least three poorly controlled cardiovascular risk factors, including diabetes, arterial hypertension, and obesity. Although our study did not allow associations, the high prevalence of cardiovascular risk factors in the patients that have SHTG-AP is similar to the data of international studies, which emphasizes the importance of lifestyle modification and optimization of the control of risk factors as the mainstay of therapeutic measures for HTG-AP [24].

Regarding the limitations of this study, there are some of them that should be highlighted. First, as this is a single-center study with a small sample, strong conclusions cannot be drawn, particularly because of the lack of statistical significance. Another major statistical limitation is that there can be false-positive results like over-estimation of the magnitude of an association. Finally, with the database of this work, it was not possible to identify the primary causes of HTG using the Fredrickson classification or obtain a thorough family history of lipid abnormalities.

## Conclusions

Patients with severe HTG are at a higher risk of developing AP. A number of comorbidities, cardiovascular risk factors, and baseline TG levels are associated with this increased incidence. Patients with severe HTG are frequently underdiagnosed, and their treatment is difficult because the need for a change of lifestyle is the foundation, which is difficult to attain and maintain. Additionally, the medication is frequently expensive and multiple. It is important to improve patient and physician education and institute more effective and appropriate interventions that effectively reduce TG levels. There is also a need to better define optimal approaches to treat SHTG. This study adds value to the existing data by helping to further define the clinical and patient characteristics with SHTG, their major complications and the best treatment to implement. Further research is required to guide future therapies and guidelines for this important clinical entity.
